# Impact of Hypoxic-Ischemic Encephalopathy on Visual Outcomes and Brain MRI Findings in Pediatric Patients: A Retrospective Observational Study From Northeast India

**DOI:** 10.7759/cureus.100056

**Published:** 2025-12-25

**Authors:** Damaris Magdalene, Ganesh Kuri, Manjisa Choudhury, Akanksha Waldia, Vidushi Dixit

**Affiliations:** 1 Pediatric Ophthalmology, Sri Sankaradeva Nethralaya, Guwahati, IND; 2 Occulopalsty, Neuro Ophthalmology, Sri Sankaradeva Nethralaya, Guwahati, IND; 3 Research, Sri Sankaradeva Nethralaya, Guwahati, IND; 4 Ophthalmology, Sri Sankaradeva Nethralaya, Guwahati, IND

**Keywords:** hypoxic-ischemic encephalopathy, mri brain, optic atrophy, pediatric neuro-ophthalmology, visual impairment

## Abstract

Objective: Hypoxic-ischemic encephalopathy (HIE) remains a significant cause of neonatal morbidity and mortality, often resulting in long-term neurological and visual impairment. This study aimed to identify the perinatal risk factors associated with HIE and evaluate the correlation between MRI abnormalities and visual outcomes in affected children.

Methodology: A retrospective observational study was conducted among 40 pediatric patients with MRI-confirmed HIE at Sri Sankaradeva Nethralaya, Guwahati, India. Demographic, perinatal, and neonatal variables were analyzed along with ophthalmic findings, including visual acuity and fundus evaluation. Statistical analysis was performed using SPSS v. 25 (IBM Corp., Armonk, NY). Chi-square, correlation, and logistic regression analyses were performed, with *P* < 0.05 considered statistically significant.

Results: The mean age was 2.9 ± 1.66 years, with a male predominance (30, 75%). Preterm birth (18, 45%) and low birth weight (20, 50%) were common and significantly associated with HIE (χ² = 6.27, *P* = 0.02; χ² = 5.18, *P* = 0.03). Neonatal hypoxia (15, 37.5%) strongly correlated with cortical MRI injury (χ² = 4.11, *P* = 0.04). MRI showed cortical lesions in 25 (62.5%), periventricular white matter changes in 10 (25%), and basal ganglia lesions in 5 (12.5% ). Visual impairment was observed in 21 (52.5%), predominantly Category IV blindness. Cortical involvement showed a significant correlation with the severity of visual impairment (Spearman’s ρ = 0.61, *P* = 0.001). Optic atrophy was the most common ocular abnormality (18, 45%) and demonstrated a strong association with cortical lesions (χ² = 8.09, *P* = 0.005). Multivariable logistic regression revealed that cortical MRI lesions (odds ratio (OR) = 5.74; 95% confidence interval (CI): 1.46-22.6; *P* = 0.01) and neonatal hypoxia (OR = 3.18; 95% CI: 1.02-9.87; *P* = 0.046) were independent predictors of severe visual impairment.

Conclusions: Preterm birth, low birth weight, and neonatal hypoxia were major contributors to HIE-related visual and neurological morbidity. Cortical MRI could serve as a key prognostic marker for visual outcomes. Early ophthalmologic screening and multidisciplinary management are critical for improving long-term outcomes.

## Introduction

Hypoxic-ischemic encephalopathy (HIE) is the outcome of varying degrees of brain injury resulting from partial or complete oxygen deprivation, which disrupts or halts cerebral blood flow. This may lead to a wide spectrum of neurological disabilities that a child has to face throughout their lifetime. The disabilities could include developmental delays, intellectual disabilities, cognitive impairment, visual and auditory problems, epilepsy, and cerebral palsy, among others.

Despite significant advancements in perinatal care, HIE remains a global health burden with reported incidences ranging from 1-3 per 1,000 live births in developed countries to 2.3-30.6 per 1,000 live births in developing nations [1,2,3]. A range of antenatal, intrapartum, and neonatal factors contribute to the development of HIE. However, in most cases, the exact cause remains unidentified [4].

The antenatal contributors include maternal age over 35 years, nulliparity, intrauterine growth restriction, chorioamnionitis, and maternal infections. These factors are linked to reduced placental function and complications during labor [4,5]. Furthermore, events leading to acute asphyxia during birth, such as antepartum hemorrhage due to uterine rupture, abruptio placenta, or placenta previa, as well as cord prolapse, tight nuchal cord, maternal shock or death, maternal hypotension, premature rupture of membranes, breech presentation, and shoulder dystocia, represent intrapartum risk factors for HIE development [6].

There is also a correlation between the term of a baby (gestational age at birth) and the risk of HIE. It is more commonly associated with term and post term infants, and the risk is higher in this group because the brain undergoes significant development during the last trimester, and the demand for oxygen and nutrients increases. However, concomitant morbidities in premature infants-respiratory distress syndrome, necrotizing enterocolitis, and bronchopulmonary dysplasia can also contribute to the development of HIE [1,7]. The predictors of perinatal HIE include abnormal fetal heart rate tracings, poor umbilical cord gases [8], low Apgar scores [7], the presence of meconium-stained fluid [9], or the need for early respiratory support [10].

The diagnosis often involves a combination of clinical features and neuroimaging. The diagnostic criteria established by the American College of Obstetricians and Gynecologists (ACOG) include a Apgar score of <5 at 5 and 10 minutes, umbilical artery pH < 7 or base deficit ≥ 12 mmol/L, along with evidence of acute brain injury as detected by magnetic resonance imaging (MRI) or magnetic resonance spectroscopy (MRS) within the first week of life and multisystem organ failure within the first 48 hours of life [11]. The ACOG criteria provide a structured diagnostic guidance and are used to define intrapartum asphyxia-related neonatal encephalopathy. Our cohort consisted of pediatric patients presenting beyond the neonatal age group. We have not applied ACOG criteria prospectively, but for our retrospective study, the cases were included based on prior documentation along with objective neuroimaging evidence available at present. The perinatal markers aligned with ACOG criteria, like a low APGAR score, history of perinatal resuscitation, and respiratory support, were extracted from records and used to select cases for our study.

Recognizing the multifactorial nature of HIE, this study was designed to evaluate perinatal risk factors associated with HIE and to comprehensively characterize central nervous system and visual manifestations, with particular emphasis on MRI findings and graded visual impairment in affected children.

## Materials and methods

Study design

This retrospective observational study included pediatric patients diagnosed with HIE between June 1, 2023, and May 31, 2024, at Sri Sankaradeva Nethralaya, Guwahati.

Ethical clearance

Ethical approval was obtained from the Institutional Ethics Committee in May 2023. IEC Approval Number: SSN/IEC/2023/04; Approval Date: May 25, 2023.

Inclusion criteria

Pediatric patients (1 month to 12 years) with clinical and radiological (CT or MRI) confirmation of HIE, and with complete ophthalmic examination and CT or MRI brain reports available, were included in the study.

Exclusion criteria

The exclusion criteria included patients with incomplete clinical data or with genetic or metabolic diseases. Inconsistent data were eliminated from the study to avoid further inaccuracies.

Data collection

The clinical and demographic data were retrieved from electronic medical records of the hospital. The variables analyzed included age at diagnosis, gender, birth weight, gestational age, and neonatal complications such as seizures, hypoglycemia, sepsis, hypoxia, dyselectrolytemia, neonatal jaundice, and hypothyroidism.

Ophthalmic assessment

The visual acuity and fundus findings were recorded for all patients. Visual disability was categorized based on the best-corrected visual acuity (BCVA) and visual field in the better and worse eye, following the standard classification (Category 0-IV) from the Gazette of India, Extraordinary Part II, Section 3, Subsection i (Ministry of Social Justice and Empowerment notification dated December 23, 2011) [12].

Category 0 represented mild or no visual impairment, defined as a BCVA of 6/9 to 6/18 in the better eye and 6/24 to 6/36 in the worse eye (20% disability). Category I represented moderate visual impairment, with a BCVA of 6/18 to 6/36 in the better eye and 6/60 to Nil in the worse eye (40% disability). Category II corresponded to severe visual impairment, defined as a BCVA of 6/40 to 4/60 in the better eye, or a visual field of 10°-20°, with the worse eye ranging from 3/60 to Nil (75% disability). Category III represented profound visual impairment, with a BCVA of 3/60 to 1/60 in the better eye, or a visual field ≤ 10°, and finger counting at 1 ft to Nil in the worse eye (100% disability). Category IV denoted total blindness, characterized by finger counting at 1 ft to Nil or a visual field ≤ 10° in both eyes (100% disability). These categories were used to classify the severity of visual loss among the study participants.

Neuroimaging

MRI brain findings were reviewed to identify cortical, periventricular white matter or basal ganglia involvement.

Statistical analysis

Categorical variables were expressed as frequency and percentages of patients, continuous variables were expressed as mean ± standard deviation or median. The Chi-square test or Fisher's exact test was used to assess associations. Correlation and logistic regression tests were also used. An alpha level of 5% was applied; that is, any *P*-value < 0.05 was considered significant. Data were analyzed using the statistical software SPSS version 25 (IBM Corp., Armonk, NY).

## Results

Demographic characteristics

As a retrospective observational study, the sample size was determined by the total number of eligible HIE cases with complete ophthalmic and MRI data during the study period. A total of 40 pediatric patients with HIE were included, comprising 30 males (75%) and 10 females (25%), yielding a male-to-female ratio of 3:1. The mean age at presentation was 2.9 ± 1.66 years (range: 4 months to 12 years). Most children (25, 62.5%) presented between one and five years of age. There was no statistically significant difference in age distribution between sexes (*P* = 0.42) (Table 1).

**Table 1:** Demographic and perinatal characteristics of children with HIE. *Statistically significant (*P* < 0.05). The mean age at presentation was 2.9 ± 1.66. SD, standard deviation; χ², chi-square test; HIE, hypoxic-ischemic encephalopathy

Parameter	Category	Frequency (*n*)	Percentage (%)	Statistical test	*P*-value
Age at presentation (years)	<1 year	9	22.5	-	-
	1-5 years	25	62.5		
	>5 years	6	15.0		
Gender	Male	30	75.0	χ² = 1.92	0.42
	Female	10	25.0		
Gestational age	Preterm (<37 weeks)	18	45.0	χ² = 6.27	0.02*
	Term (≥37 weeks)	22	55.0		
Birth weight	<2.5 kg	20	50.0	χ² = 5.18	0.03*
	≥2.5 kg	20	50.0		
Neonatal complications	Hypoxia	15	37.5	χ² = 4.11	0.04*
	Jaundice	14	35.0		
	Seizures	12	30.0		
	Hypoglycemia	5	12.5		
	Sepsis	4	10.0		

Perinatal and neonatal risk factors

Gestational Age

**Figure 1:**
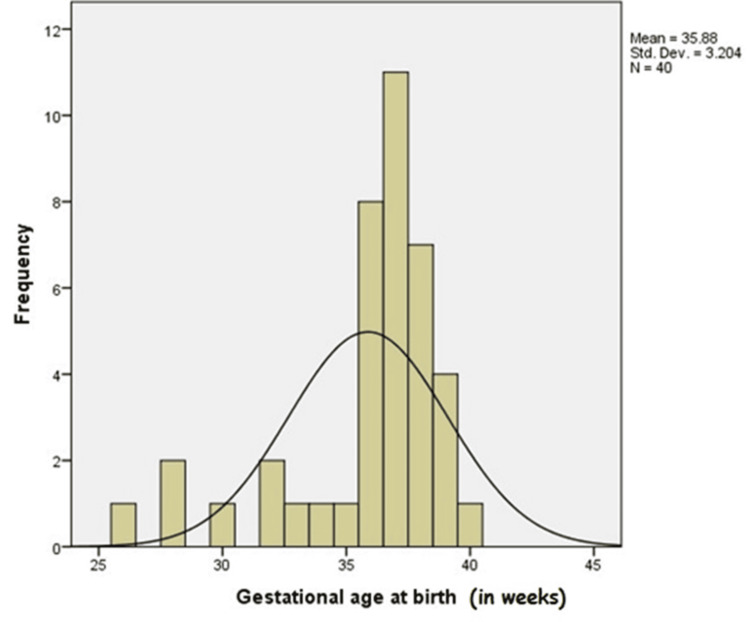
Frequency histogram correlating the gestational age at birth with HIE. HIE, hypoxic-ischemic encephalopathy

Gestational age was included as one of the study parameters. Neonates were classified at birth as preterm (32-36 weeks), term (37-42 weeks), or post-term (>42 weeks). The mean gestational age was 35 ± 3.20 weeks. Figure 1 depicts the frequency histogram correlating the gestational age at birth with HIE.

Birth Weight

**Figure 2:**
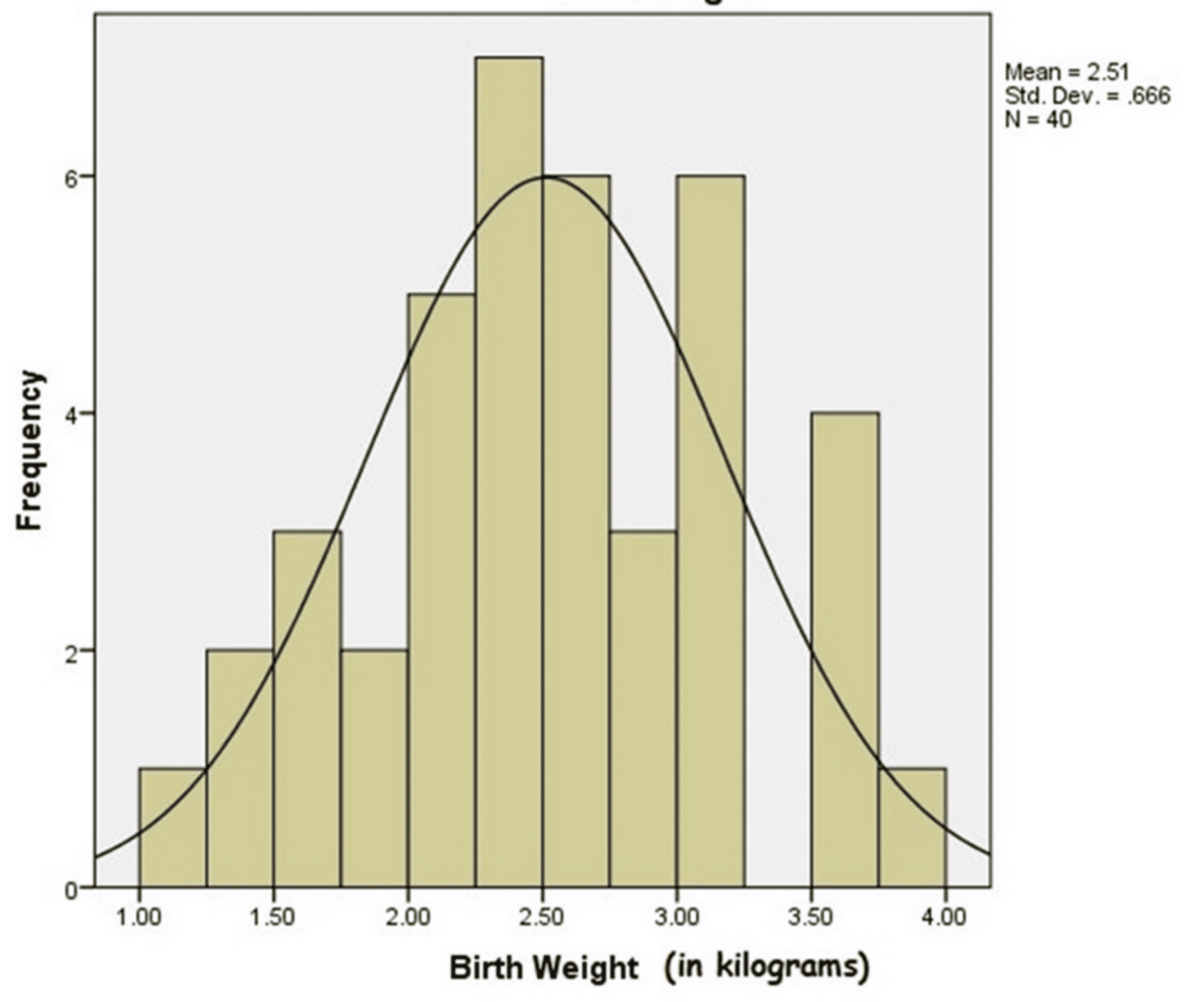
Frequency histogram depicting the correlation between birth weight and HIE. HIE, hypoxic-ischemic encephalopathy

The birth weight of neonates was also studied with respect to the development of HIE. The neonatal birth weight was categorized as low birth weight (1,500-2,499 g) and normal birth weight (2,500-4,000 g). The mean birth weight was 2.51 ± 0.66 kg. Figure 2 depicts the frequency histogram correlation between birth weight and HIE.

Preterm birth was observed in 18 children (45%), and 20 (50%) had low birth weight (<2.5 kg). Neonatal hypoxia was the most frequent complication (37.5%), followed by jaundice (35%) and seizures (30%). Other neonatal factors included hypoglycemia (12.5%) and sepsis (10%).

Preterm birth and low birth weight were significantly associated with the presence of HIE compared with term and normal-weight neonates (χ² = 6.27, *P* = 0.02). Similarly, hypoxia at birth was significantly correlated with MRI evidence of cortical injury (χ² = 4.11, *P* = 0.04). The demographic and perinatal characteristics of children with HIE are detailed in Table 1.

Other complications and probable risk factors that could lead to the development of HIE were also identified from the medical records and correlated with HIE development. Seizure, hypoglycemia, hypoxia, sepsis, neonatal jaundice, cerebral palsy, hydrocephalus, electrolyte imbalance, and hypothyroidism were the variables studied (Table 2).

**Table 2:** Details of neonatal complications in patients with HIE. HIE, hypoxic-ischemic encephalopathy

Neonatal complications	Frequency	Percentage
Seizure	12	30.00
Hypoglycemia	5	12.50
Hypoxia	15	37.50
Sepsis	4	10.00
Neonatal jaundice	14	35.00
Cerebral palsy	3	7.50
Hydrocephalus	1	2.50
Electrolyte imbalance	1	2.50
Hypothyroidism	2	5.00

Visual outcomes

Visual disability was classified according to the Gazette of India (with best correction). Blindness was defined as total absence of sight, characterized by finger counting at 1 ft to nil or a visual field of ≤10°. For reporting comparability, the World Health Organization/International Classification of Diseases (WHO/ICD) defines blindness as presenting visual acuity in the better eye of <3/60, which is further graded into categories 3-5 according to WHO/ICD thresholds. Owing to slight differences in the categorization of visual impairment and blindness, visual disability was categorized using the Gazette of India classification, as it is the standard framework used for certification and rehabilitation services in our setting. WHO/ICD is a severity classification, but the Gazette of India provides a benefits-oriented disability certification system.

Since the main reason for presenting at the tertiary eye care center was complaints related to the visual system, the patients were categorized according to the severity of diminution of vision and various morphologies and pathologies, and were evaluated with the help of fundus examination.

Visual Acuity

The visual acuity of the children was assessed and categorized based on the disability criteria into four categories as detailed in Table 3. The mean visual acuity among affected children was 1.85 ± 0.32 logMAR.

**Table 3:** Visual outcomes of patients with HIE. *Statistically significant (*P* < 0.05). χ², chi-square test; HIE, hypoxic-ischemic encephalopathy

Disability category	Frequency	Percentage	Statistical test	P-value
Category 0	5	12.5	χ² = 10.32	0.002*
Category 1	5	12.5		
Category II	2	5.0		
Category IIIC (low vision)	2	5.0		
Category IIIE (low vision)	2	5.0		
Category IVA blindness	3	7.5		
Category IVB blindness	21	52.5		
Total	40	100.0		

Fundus Evaluation

On fundus evaluation, optic atrophy was found in 18 (45%), temporal pallor in 11 (27.5%), and normal discs in 10 (25%). One child (2.5%) showed concurrent optic atrophy with nystagmus. The proportion of optic atrophy was significantly higher in those with cortical lesions compared to those without (χ² = 8.09, *P* = 0.005) (Table 4).

**Table 4:** Fundus evaluation of patients with HIE. *Statistically significant (*P* < 0.05). χ², chi-square test; HIE, hypoxic-ischemic encephalopathy

Status of optic disc	Frequency	Percentage	Statistical test	P-value
Optic atrophy	18	45.0	χ² = 8.09	0.005*
Optic atrophy, nystagmus	1	2.5		
Temporal pallor	11	27.5		
Normal	10	25.0		
Total	40	100.0		

Neuroimaging findings

HIE primarily refers to brain damage that is caused by hypoxia. The anatomical and pathological damage to the brain matter was studied with the help of MRI. The details of MRI findings are mentioned in Table 5.

**Table 5:** Specific brain regions affected in patients with HIE. HIE, hypoxic-ischemic encephalopathy

Specific brain regions affected in HIE patients	Frequency	Percentage
Basal ganglia	5	12.5
Cerebral cortex	25	62.5
Periventricular white matter (pvl)	10	25.0
Total	40	100.0

A strong positive correlation was noted between the extent of cortical lesions and the severity of visual impairment (Spearman’s ρ = 0.61, *P* = 0.001) (Table 6).

**Table 6:** Correlation between MRI lesion and vision. *Statistically significant (*P* < 0.05). ρ, Spearman correlation coefficient

Category	Statistical test	*P*-value
Cortical lesion and visual impairment	Spearman’s ρ = 0.61	0.001*
White matter lesion and visual impairment	Spearman’s ρ = 0.18	0.27
Basal ganglia lesion and visual impairment	Spearman’s ρ = 0.15	0.33

Correlation between MRI findings and visual parameters

As this was a retrospective observational study in a pediatric cohort, the MRI pattern classification was based on radiology report descriptors and the region(s) involved, and each scan was categorized by the predominant region of injury as: (1) cerebral cortex/cortico-subcortical (including watershed/parasagittal cortical injury), (2) periventricular white matter injury consistent with PVL, and (3) basal ganglia/thalamic involvement. These categories were defined using descriptions of MRI patterns of hypoxic-ischemic brain injury.

The cortical involvement on MRI demonstrated a significant association with both visual acuity category and fundus abnormalities (χ² = 10.32, *P* = 0.002) (Table 3). In contrast, periventricular and basal ganglia lesions did not show statistically significant correlation with visual outcomes (*P* = 0.27 and *P* = 0.33, respectively) (Table 6).

Multiple logistic regression analysis identified cortical MRI involvement (odds ratio (OR) = 5.74; 95% confidence interval (CI): 1.46-22.6; *P* = 0.01) and neonatal hypoxia (OR = 3.18; 95% CI: 1.02-9.87; *P* = 0.046) as independent predictors of severe visual impairment (Table 7).

**Table 7:** Logistic regression analysis (multivariate). *Statistically significant (*P* < 0.05). OR, odds ratio; CI, confidence interval

Category	OR	95% CI	*P*-value
Cortical MRI lesion	5.74	1.46-22.6	0.01*
Neonatal hypoxia	3.18	1.02-9.87	0.046*

## Discussion

This retrospective observational study evaluated perinatal characteristics, visual outcomes, and neuroimaging findings in children diagnosed with HIE who visited the outpatient department of a tertiary eye care setting in Northeast India. The study highlighted a high burden of visual impairment and demonstrated a significant association between cortical MRI abnormalities and the severity of visual disability.

A marked predominance of male children (30, 75%) was observed in the study cohort, consistent with previous studies by Wang et al. [13] and Jarvis et al. [14].

Preterm birth and low birth weight emerged as key contributors, both of which were significantly linked to HIE (*P* = 0.02 and *P* = 0.03). The mean gestational age of our cohort was 35 ± 3.20 weeks. A study by Futraful et. al. revealed that post-term gestational age (≥ 36 weeks) was a risk factor for the development of HIE [15]. Furthermore, 20 (50%) of our study cohort had low birth weight. Studies have reported that the infants who developed HIE had significantly lower birth weight than the infants who did not develop HIE [16,17]. Low-birth-weight and premature infants were associated with an increased risk of moderate or severe HIE [18]. Low neonatal birth weight and premature conditions are known to predispose the immature brain to hypoxic-ischemic injury due to incomplete cerebral autoregulation and reduced metabolic reserve [13].

More than half of the children (21, 52.5%) in this cohort had severe visual impairment categorized as category IV blindness, with only 10 (25%) showing normal optic disc morphology on fundoscopic examination. Optic atrophy was the most common abnormality (18, 45%), reflecting the high proportion of post-ischemic damage to the optic nerve and associated structures. The presence of temporal pallor (11, 27.5%) and nystagmus (1, 2.5%) further highlighted the spectrum of visual sequelae in HIE, ranging from subtle abnormalities to profound visual impairment. Thus, visual dysfunction was a predominant finding with HIE, corroborating the following earlier studies. In a single-center retrospective study of 57 neonates with a diagnosis of HIE, with respect to visual outcomes, a positive correlation was reported between severe HIE and visual pathway dysfunction [19]. In another study, a positive association was reported between the findings of visual assessment performed at the age of five months and neurodevelopmental outcome at the age of two years among children with sustained hypoxic-ischemic insults [20]. These findings emphasized the need for early ophthalmologic screening in children suffering from HIE to ensure timely intervention, rehabilitation, and appropriate visual aids. The degree of visual derangement could also be viewed as a probable prognostic marker for the level of ischemic insult to the brain.

Cortical lesions were the most frequent MRI abnormality identified in this study and demonstrated a strong positive correlation (ρ = 0.61, *P* = 0.001) with the severity of visual impairment. A moderate reduction in cerebral blood flow in the gestational period due to hypoxia in patients with HIE leads to shunting of blood flow from the anterior circulation to the posterior circulation, to maintain adequate perfusion of the cerebellum, brainstem, and basal ganglia. As a result, the damage is restricted to the cerebral cortex and watershed areas of the cerebral hemispheres. Acute hypoxia could cause an abrupt decrease in cerebral blood flow, which could impart injury to the basal ganglia and thalamus [6]. Basal ganglia involvement was less common (12.5%), consistent with patterns typically seen in acute profound hypoxic insults. Periventricular white matter changes were observed in a subset of patients (10, 25%), reflecting the vulnerability of developing oligodendrocytes in preterm and low birth weight infants. These patterns were consistent with studies that had linked previous neuroimaging MRI findings to HIE [21]. However, it is important to emphasize that this study demonstrated correlation rather than direct causation. While cortical involvement was independently associated with severe visual impairment on multivariable analysis, the retrospective design of the study would preclude definitive causal inference.

Notably, further multivariate logistic regression analysis identified cortical MRI lesions (OR = 5.74) and documented neonatal hypoxia (OR = 3.18) as independent predictors of severe visual impairment. These findings emphasized the prognostic utility of neuroimaging in children with HIE and suggested that MRI patterns could help stratify patients at higher risk for long-term visual disability.

Novelty and relevance

This study finding contributes to the existing literature in several important ways. First, it systematically correlates MRI injury patterns with graded visual disability rather than isolated ophthalmic findings. Second, it applies the Gazette of India visual disability classification, enhancing translational relevance for disability certification and rehabilitation planning within the Indian healthcare system. Finally, the study provides data from Northeast India, a region that remains underrepresented in studies examining visual outcomes in HIE.

Limitations of this study

The retrospective, single-center design and modest sample size could limit generalizability. The sample size was limited to all available eligible cases within the study timeframe; therefore, no prior power calculation was feasible. Nonetheless, the strength of association between neuroimaging patterns and visual outcomes could provide a strong rationale for larger multicenter longitudinal studies.

Clinical implications

Integrating ophthalmic assessment into neonatal HIE follow-up protocols would be vital. The early detection of cortical and optic nerve pathology could allow for prompt visual intervention, neuro-rehabilitation, and family counselling to improve quality of life.

## Conclusions

HIE remains a significant contributor to visual and neurological morbidity in children. In this retrospective study, preterm birth, low birth weight, and documented neonatal hypoxia were commonly observed among affected children and were significantly associated with adverse visual outcomes.

The cortical involvement detected on MRI showed a strong positive correlation with the severity of visual impairment, highlighting the potential value of neuroimaging in risk stratification and clinical assessment. However, given the retrospective observational nature of the study, these findings are to be interpreted as associative rather than causal.

This study is among the first to systematically categorize visual disability in children with HIE in India and correlate these functional outcomes with MRI findings. These results add to the existing literature by emphasizing the importance of integrated neuro-ophthalmic evaluation. A multidisciplinary approach involving neonatologists, neurologists, and ophthalmologists would be essential to address the complex clinical needs of these patients. Future prospective and longitudinal studies are warranted to further elucidate causal pathways and long-term visual outcomes.
